# Towards a more efficient diabetes control in primary care: six-monthly monitoring compared with three-monthly monitoring in type 2 diabetes - The EFFIMODI trial.  Design of a randomised controlled patient-preference equivalence trial in primary care

**DOI:** 10.1186/1471-2296-11-35

**Published:** 2010-05-11

**Authors:** Paulien R Wermeling, Maureen van den Donk, Kees J Gorter, G Ardine de Wit, Yolanda van der Graaf, Guy EHM Rutten

**Affiliations:** 1 Julius Center for Health Sciences and Primary Care, University Medical Center Utrecht, Utrecht, the Netherlands; 2 National Institute of Public Health and the Environment, Bilthoven, the Netherlands

## Abstract

**Background:**

Scientific evidence for the frequency of monitoring of type 2 diabetes patients is lacking. If three-monthly control in general practice could be reduced to six-monthly control in some patients, this would on the one hand reduce the use of medical services including involvement of practice nurses, and thus reduce costs, and on the other hand alleviate the burden of people with type 2 diabetes. The goal of this study is to make primary diabetes care as efficient as possible for patients and health care providers. Therefore, we want to determine whether six-monthly monitoring of well-controlled type 2 diabetes patients in primary care leads to equivalent cardiometabolic control compared to the generally recommended three-monthly monitoring.

**Methods and design:**

The study is a randomised controlled patient-preference equivalence trial. Participants are asked if they prefer three-monthly (usual care) or six-monthly diabetes monitoring. If they do not have a preference, they are randomised to a three-monthly or six-monthly monitoring group. Patients are eligible for the study if they are between 40 and 80 years old, diagnosed with type 2 diabetes more than one year ago, treated by a general practitioner, not on insulin treatment, and with HbA1c ≤7.5%, systolic blood pressure ≤145 mmHg and total cholesterol ≤5.2 mmol/l. The intervention group (six-monthly monitoring) will receive the same treatment with the same treatment targets as the control group (three-monthly monitoring). The intervention period will last one and a half year. After the intervention, the three-monthly and six-monthly monitoring groups are compared on equivalence of cardiometabolic control. Secondary outcome measures are HbA1c, blood pressure, cholesterol level, Body Mass Index, smoking behaviour, physical activity, loss of work due to illness, health status, diabetes-specific distress, satisfaction with treatment and adherence to medications. We will use intention-to-treat analysis with repeated measures. For outcomes that have only baseline and final measurements, we will use ANCOVA. Depending on the results, a cost-minimisation analysis or an incremental cost-effectiveness analysis will be done.

**Discussion:**

This study will provide valuable information on the most efficient control frequency of well-controlled type 2 diabetes patients in primary care.

**Trial registration:**

Current Controlled Trials ISRCTN93201802

## Background

At the end of 2007 more than 660.000 people were diagnosed with type 2 diabetes in the Netherlands [1]. The number of type 2 diabetes patients is still increasing [1], and also their use of health care facilities. Furthermore, the overall workload of general practitioners is increasing [2]. The current guideline on type 2 diabetes in primary care in the Netherlands advises to monitor type 2 diabetes patients four times a year [3], but this advice is not evidence-based. Three quarterly controls are done by the practice nurse and the general practitioner is advised to perform the annual check-up. Comparing 15 diabetes guidelines in 13 countries, the advised frequency of monitoring HbA1c ranged from one to four times a year and monitoring blood pressure ranged from one to six times a year [4]. It is obvious that the workload for healthcare professionals will differ significantly, depending on the guideline that is followed.

A retrospective, observational study in Spain demonstrated that the improvement in glycaemic control over time in patients with type 2 diabetes in general practice was not related to the number of visits to the general practitioner, but to changes in treatment [5]. More evidence on the desired frequency of type 2 diabetes controls in general practice is lacking.

A randomised equivalence trial in Canada compared blood pressure control, adherence to treatment and patient satisfaction in patients with treated hypertension followed up by their family physicians every three or six months. Patients with follow-up every three months achieved the same levels of blood pressure control, adherence to treatment and patient satisfaction compared to patients with follow-up every six months [6].

If, in accordance with the hypertension example, the contact time with the diabetes team in well-controlled type 2 diabetes patients could also be reduced up to 50% without deteriorating their quality of care, this could reduce the patient burden as well as induce savings on direct medical costs and relieve the workload of practice nurses.

Therefore we designed the EFFIcient MOnitoring of DIabetes (EFFIMODI) study, aiming to make primary diabetes care as efficient as possible for patients and health care providers. We hypothesise that six-monthly monitoring of well-controlled patients with type 2 diabetes in primary care results in equivalent cardiometabolic control as the currently recommended three-monthly monitoring, with less costs.

## Methods and design

### Study design

**Figure 1:**
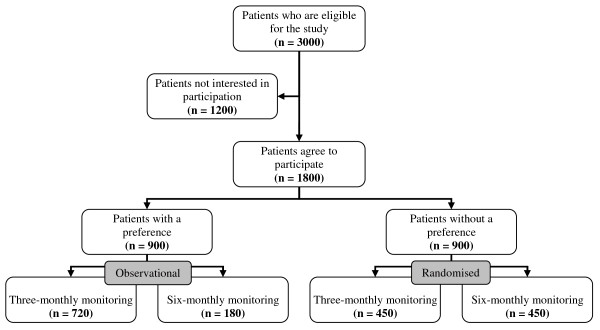
**Participant flowchart**. The participant flowchart with the expected number of patients, based on a small survey.

The study has been designed as a randomised controlled patient-preference equivalence trial. In practice this design means that participants are asked if they prefer three-monthly (usual care) or six-monthly diabetes monitoring. If they do not have a preference, they are randomised to a three-monthly or six-monthly monitoring group. This will result into four study groups (see Figure 1).

We chose to conduct a patient-preference trial because of two reasons [7]. First, it gives us the possibility to compare the relationship between patients' preference for a frequency of diabetes control and the study outcomes. Second, more people will participate in the study, as people can choose not to be randomised; they can be included in the so-called 'observational' study arm of their choice. Doing so, we will have information about people who are not randomised and this will help in generalising the results. In conducting a patient-preference trial we will avoid selection and probably also drop-out after randomisation.

We chose to conduct an equivalence trial, because we want to assess whether six-monthly monitoring results in equal cardiometabolic control compared to the current frequency of control [8]. Since we expect that six-monthly monitoring will not give better cardiometabolic control than three-monthly monitoring, we did not choose a non-inferiority trial. The intervention period will last one and a half year, so patients will be seen either seven or four times during the intervention period.

The Medical Research Ethics Committee of the University Medical Center Utrecht has approved the study protocol (Protocol number: 08-453).

### Study population

Patients are eligible for the study if they are between 40 and 80 years old, diagnosed with type 2 diabetes for more than a year, treated by their general practitioner, not on insulin treatment and overall well-controlled, defined as having HbA1c ≤7.5% and systolic blood pressure ≤145 mmHg and total cholesterol ≤5.2 mmol/l. These HbA1c, systolic blood pressure and total cholesterol values are a little higher than the Dutch target values (HbA1c ≤7.0%, systolic blood pressure ≤140 mmHg and total cholesterol ≤4.5 mmol/l [3]). We decided to choose more liberal values to create a larger target population to be randomised. According to the stricter target values only 18.9% of all type 2 diabetes patients fulfilled these values [9]. Because of the minimal difference with the target values, we assume that 20% of the type 2 diabetes patients will fulfil the inclusion criteria.

### Recruitment of practices and patients

We will approach the boards of several health care groups to recruit general practitioners. These health care groups have a central database in which all type 2 diabetes patients are recorded. In this database all determinants that are needed for the selection of study patients are included (see **study population **for the inclusion criteria). The health care groups will ask all their affiliated general practitioners to participate in the study. If a general practitioner wants to participate he will obtain the selection of patients, according to the inclusion criteria, from the health care group. The general practitioner sends an information letter as well as an informed consent form to the selected patients.

Patients who want to participate have to fill in the informed consent form. The participants are asked whether they strongly prefer three-monthly (current care) or six-monthly diabetes control or whether they have no preference. Patients with a preference will enter the 'observational arm'. If participants do not have a strong preference for the frequency of care, they will be randomised.

### Randomisation and blinding

Participants without a preference for either three-monthly or six-monthly monitoring are randomised into one of the two randomised study arms in a 1:1 ratio: a control group that will receive current care, comprising diabetes control once per three months, and an intervention group that will receive diabetes control once per six months. In both randomised groups the extensive annual check-up will be done by the general practitioner. The treatment targets, therapeutic algorithms and life style advices will remain unchanged and do not differ between the intervention and control groups.

Randomisation is generated at the patient level by a computerised random-number generator at the Julius Center. Participants are randomised before baseline data are collected. Since this is a pragmatic trial, it is not necessary to blind participants and general practitioners for the treatment allocation. However, the laboratory technicians who measure HbA1c and cholesterol are not aware of study participation of the patients.

### Outcomes

The primary outcome measure is the percentage of people that remains under good cardiometabolic control, defined as having HbA1c ≤7.5% and systolic blood pressure ≤145 mmHg and total cholesterol ≤5.2 mmol/l. Secondary outcome measures are HbA1c, blood pressure, cholesterol, Body Mass Index, fasting blood glucose, smoking behaviour, physical activity, loss of work due to illness, health status, diabetes-specific distress, satisfaction with treatment and adherence to medications. These outcomes are collected either from the medical records or from a patient questionnaire.

#### Medical records

Biomarkers (HbA1c, cholesterol, fasting blood glucose) and anthropometric variables (blood pressure, Body Mass Index) will be collected from the general practitioners' computerised information system. Information on the most recently measurements of the biomarkers and anthropometric variables before the study, medical history, medication use before the study and all measurements performed during the first EFFIMODI visit will be collected just after the start of the study. All measurements during the follow-up period will be collected after the end of the follow-up period. The same applies to the number of diabetes and non-diabetes related visits to the general practitioner and (differences in) medication use during the study.

#### Questionnaires

The participants will be asked to complete an extensive questionnaire before (t = 0) and after (t = 18 months) the intervention period. This questionnaire comprises general background information on age, gender, ethnicity, education, smoking, physical activity, occupation and loss of work due to illness, health status, diabetes-specific distress and satisfaction with diabetes treatment.

Current smoking is measured as the number of cigarettes per day or the number of cigars per week. Smoking in the past is measured the same way. Also the number of pack years is recorded. Physical activity is measured with the Short Questionnaire to Assess Health-enhancing physical activity (SQUASH) [10]. The SQUASH is a reliable and valid questionnaire to measure the level of physical activity in an adult population. The questionnaire was designed to give an indication of the habitual activity level. Information on light (range: 2-4 Metabolic Equivalent of Task (MET)), moderate (range: 4-6.5 MET) and vigorous (>6.5 MET) intensity physical activities will be obtained. Physical activity will be expressed in minutes per week and in a total activity score. The total activity score will be calculated by multiplying the minutes per week by the actual MET score of the specific activity (MET/min/week).

Occupation and loss of work due to illness are measured with the Short Form Health and Labour Questionnaire (SF-HLQ) [11]. The SF-HLQ consists of three parts: absenteeism from paid work, production losses without absenteeism from paid work and hindrance in the performance of paid and unpaid work. Data derived with this questionnaire will be used to calculate costs of productivity losses, should incremental cost-effectiveness be merited at closure of the trial (see **economic evaluation**).

Two questionnaires are used to measure health status: Short-Form 36 (SF-36) [12] and EQ-5D [13]. The SF-36 generates a profile of scores on eight dimensions of health. These dimensions are: (1) physical functioning; (2) limitations due to physical difficulties (physical role functioning); (3) bodily pain; (4) social functioning; (5) mental health; (6) limitations due to emotional difficulties (emotional role functioning); (7) vitality; and (8) general health perception. For all eight dimensions an average score for all items in the scale is calculated, with a range from 0 (least favourable health state) to 100 (most favourable health state). Two summary scales for mental and physical functioning can be calculated as well. The SF-36 is validated in the Dutch population [14].

The EQ-5D is a generic questionnaire, consisting of a Visual Analogue Scale (EQ-5D VAS) and a classification system (EQ-5D Profile) [13]. The EQ-5D Profile covers five domains of health (mobility, self-care, usual activities, pain/discomfort and anxiety/depression), each with three levels of functioning: level 1, no problems; level 2, some problems; level 3, severe problems. The EQ-5D VAS is a graded, vertical line, anchored at 0 (worst imaginable health state) and 100 (best imaginable health state). The patient is asked to mark a point on the EQ-5D VAS that best reflects his/her actual health state.

To measure diabetes-specific distress, the Problem Areas In Diabetes (PAID) questionnaire is used [15]. This is a widely recognised measure of diabetes distress, assessing the general emotional burden of diabetes and distress related to treatment, food choices and social support. The 20 items are scored on a 5-point scale yielding a sum score (0-80), with higher scores representing higher distress. The Dutch PAID scale has good convergent and discriminate validity and high internal consistency [16].

To measure satisfaction with diabetes treatment, the Diabetes Treatment Satisfaction Questionnaire (DTSQ) is used [17]. The DTSQ measures satisfaction with treatment regimen (six items), perceived frequency of hyperglycaemia (one item) and perceived frequency of hypoglycaemia (one item) over the past few weeks. The treatment satisfaction score can range from 0 (very dissatisfied) to 36 (very satisfied).

#### Economic evaluation

To be able to calculate direct health care costs, data on health care use are needed. Data concerning consultations beyond the planned monitoring consultations, medication use and referral rates to other health care professionals will be collected from the general practitioners' computerised Information System. Should this trial provide evidence that three-monthly monitoring results in better outcomes than six-monthly monitoring, an incremental cost-effectiveness analysis becomes warranted, that quantifies the additional cost related to the additional health effects. For such an economic evaluation we will use data on health care use as recorded in the information system of the general practitioners, and data recorded with the SF-HLQ and the EQ5D (see above). The EQ5D is of special importance, as utilities, and consequently, quality adjusted life years (QALYs) can be elicited using this generic questionnaire.

### Sample size calculation

The sample size is calculated on the assumption of equivalence of cardiometabolic control. Therefore, we used the formula from Jones et al. [8]: n = (2p(100-p)*(Z_(1-α)_+Z_(1-β)_)^2^)/δ^2^, where p is the overall percentage of successes to be expected if the treatments are equivalent, δ indicates the range of equivalence for the difference in percentage success rates, α is the probability of type I error and β the probability of type II error.

We assume equivalence if the two-sided 95% confidence interval (α = 0.05; Z_(1-α) _= 1.96) for the difference in cardiometabolic control between the two intervention groups is completely in the range from -5 to 5% (δ = 5). With a supposed overall percentage of success of 95% and a power of 90% (β = 0.1; Z_(1-β) _= 1.28), we need a sample size of 399 people per randomised group. Based on a small survey that was performed by the Julius Center, we assume that ~50% of the people have no preference and thus will be randomised. About the other half of the people is assumed to have a strong preference for the frequency of monitoring. Therefore, we need to include 1596 patients: 798 in the randomised arms and 798 in the observational arms.

As said earlier in the **study population **section, we assume that 20% of the type 2 diabetes patients will fulfil the inclusion criteria. With an average of 80 type 2 diabetes patients per practice, we expect that sixteen patients in each general practice will meet the eligibility criteria. Taking into account a response rate of 60%, ten patients in each practice will be willing to participate in the study: five in the observational arm and five in the randomised arm, so at least 160 general practitioners will have to be recruited. As people may drop out during the study, we will recruit 1800 patients allowing an 11% drop-out; therefore we need 180 general practitioners. Figure 1 shows the participant flowchart with the expected numbers.

### Analysis

We will use repeated measures analysis for all recorded measurements of HbA1c, blood pressure, cholesterol and fasting blood glucose to optimally use all available data. Data from the questionnaires at the start and at the end of the intervention period will be analysed using ANCOVA. Data will be analysed according to intention-to-treat. For handling missing data we will use multiple imputation [18]. After the intervention, the randomised three-monthly and six-monthly monitoring groups are compared on equivalence of cardiometabolic control.

**Figure 2:**
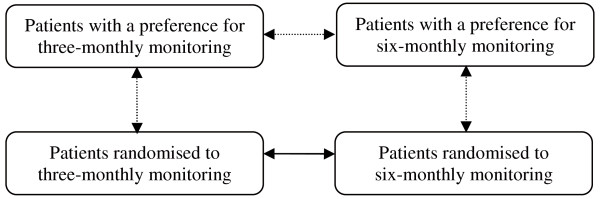
**Group comparisons**. An overview of all comparisons that will be made between the four groups. The solid arrow is the comparison to answer the main research question. The dotted arrows are other comparisons that will be made.

Since this trial is a patient preference trial, we will also compare the three-monthly preference group with the three-monthly randomised group. Depending on the number of patients opting for the six-monthly controls, we will compare this group with the six-monthly randomised group. In these analyses, we will examine determinants of preference, and we will determine risk profiles of patients. This will facilitate the applicability of the results and we can demonstrate if people who are more motivated will have better cardiometabolic control. Figure 2 provides an overview of all comparisons that will be made between the groups.

#### Economic evaluation

Should this study demonstrate equivalent outcomes with different frequencies of diabetes monitoring, the evident cost-reduction of less control visits merit the conclusion that six-monthly monitoring is the approach of first choice and a cost-minimisation analysis will be done. This is likely to provide sufficient evidence to change the frequency of monitoring in well-controlled type 2 diabetes patients in general practice.

However, should the three-monthly follow-up scheme result in a better cardiometabolic control, a cost-effectiveness analysis will be performed. If better outcomes can be realised with a higher control frequency at an associated higher cost to society, or vice versa, (somewhat) worse patient outcomes at a lower cost, the balance between costs and outcomes is of interest. Differences in the number of QALYs between groups during the study period will be assessed. The difference in treatment effect will be calculated as follows: for each group the difference between baseline and final measurements in percentage of patients with good cardiometabolic control will be determined. The absolute difference between measurements pre- and post-intervention will be taken as the intervention effect. Accordingly, differences in QALYs between groups will be calculated. The 'incremental cost-effectiveness ratio' (ICER) will be expressed as cost differences between groups divided by differences in treatment effects between groups. Confidence intervals will be determined using bootstrapping [19]. A 'cost-effectiveness acceptability curve' (CEAC) will also be drawn using the bootstrap sample. Cost-utility estimates will be derived accordingly, using QALY differences between groups as outcome measure.

## Discussion

If the results of this study will show that equivalent cardiometabolic control is achieved following six-monthly diabetes control in a sub-sample of generally well-controlled people with type 2 diabetes in general practice as compared to the usual three-monthly diabetes control, their diabetes control frequency can be reduced. This will reduce the use of medical services and direct health care costs, alleviate the burden of a substantial part of the people with type 2 diabetes and relieve the workload of diabetes nurses. If three-monthly monitoring turns out to result in a better regulation of diabetes, a cost-effectiveness analysis is necessary to estimate whether the higher costs of three-monthly follow-up balance the better patient outcomes.

The results of this study will provide valuable information for health care professionals and policy makers on cost-effectiveness of diabetes monitoring. In the case of proven cost-effectiveness, we will recommend implementing a lower control frequency for well-controlled type 2 diabetes patients.

## Competing interests

The authors declare that they have no competing interests.

## Authors' contributions

GEHMR is the principal investigator for the EFFIMODI trial. The study design and research proposal were worked out by GEHMR, MvdD, KJG and YvdG. PRW, MvdD and KJG are the trial co-ordinators. GAdW is the health economist and is involved in the cost-effectiveness analysis. PRW drafted the manuscript. All authors have corrected draft versions and approved the final manuscript.

## Pre-publication history

The pre-publication history for this paper can be accessed here:

http://www.biomedcentral.com/1471-2296/11/35/prepub
